# Sex differences in physical activity among Ghanaian patients with sickle cell disease

**DOI:** 10.11604/pamj.2019.32.63.14643

**Published:** 2019-02-05

**Authors:** Gifty Gyamah Nyante, Catherine Oppong, Emmanuel Bonney

**Affiliations:** 1 Department of Physiotherapy, School of Biomedical & Allied Health Sciences, College of Health Sciences, University of Ghana, Ghana

**Keywords:** Physical activity, physical fitness, Sickle Cell Disease, Ghanaian patients

## Abstract

**Introduction:**

Musculoskeletal pain and functional limitations experienced by patients with Sickle Cell Disease (SCD) impact their physical activity and social behaviour. Yet, we know little about physical activity behaviour in patients with SCD. The aim of this study was to investigate gender differences in physical activity, sedentary time and measures of fitness among Ghanaian adults with SCD. The study also determined the association between outcome variables in this population.

**Methods:**

Patients with SCD attending a sickle cell clinic in a tertiary hospital in Accra, Ghana participated in this cross-sectional study. Physical activity, sedentary time, body composition, flexibility and cardiovascular endurance were assessed. Demographic data were also collected by self-report.

**Results:**

Fifty three participants enrolled in the study. Of these, more than half were females (60.4%) and the average age of the participants was (M: 26.8, SD: 8.5 years). The total physical activity reported by the participants was different between males and females (t = 2.610, p = 0.012). However, there were no gender differences in sedentary time, body composition, flexibility and cardiovascular endurance. A moderately significant association was found between sedentary time and cardiovascular endurance (r = 0.437, p = 0.001).

**Conclusion:**

The findings suggest that gender differences in physical activity are apparent in patients with SCD. Investigations into the mechanisms underpinning these differences are warranted. Additionally, longitudinal observations of objectively measured physical activity may be useful to validate these results in a larger sample.

## Introduction

Physical activity is an important health-enhancing behaviour with established benefits in apparently healthy populations [1, 2] and individuals with pathological conditions [3-6]. Despite these, physical inactivity and low cardiorespiratory fitness have become a global concern probably because of the relationship between physical inactivity and non-communicable diseases [7-10]. To design effective interventions to address physical inactivity in a particular group, accurate physical activity data on the relevant population is needed [11]. Therefore, it is prudent to measure physical activity levels and to determine gender differences among patients with Sickle Cell Disease (SCD) in the Ghanaian context. Physical activity has been extensively studied in different populations [12-15]. Unfortunately, there are no documented reports on physical activity and sedentary behaviour in a sickle cell population. Thus, the most basic questions remain to be answered; Are there gender differences in physical activity among patients with SCD? Do persons with Sickle Cell Disease (SCD) engage in sufficient amount of physical activity? How does SCD influence physical activity and sedentary behaviour? What is the relationship between physical activity, sedentary time and physical fitness in individuals with SCD? Rarely has research addressed these critical questions. Therefore, primary research on physical activity is needed to increase knowledge about physical activity behaviour in this population. In addition, such data may aid the development of strategies to minimize functional limitations experienced by patients when performing activities of daily living. SCD is a genetic disorder that affects both children and adults [16-18] and is known to be more common within the Sub-Saharan Africa compared to other parts of the world [18]. In Ghana, SCD is linked to high rate of mortality and morbidity, with prevalence of about 1.6% [19]. It has been reported that 25-30% of the Ghanaian adults are carriers of the sickle cell trait and 2% of babies have SCD [20, 21]. Notable complications associated with SCD include painful crisis, infection, acute chest syndrome, priapism, stroke, renal medullary cancer and splenic infarction [16, 18, 22]. Evidently both children and adults with SCD have lower quality of life and poor functional capacity compared to those without the disease [16, 23]. Also, people with SCD are often stigmatized [20], which negatively impact their social behaviour. The numerous complications associated with SCD could be expected to have a negative impact on physical activity. Likewise, reduced physical activity may further worsen patients' risk for other non-communicable diseases. To optimize the functional capacity of adults with SCD, a multidisciplinary management strategy that gives consideration to both medical and non-medical problems should be adopted. However, this would require better understanding of the impact of the disease on known health indicators such physical activity and fitness. Since physical activity predicts functional capacity and influences quality of life, deeper insight about the physical activity behaviour of patients with SCD is necessary. There is generally lack of information on physical activity among patients with SCD worldwide. To the authors' knowledge, no study has examined gender differences among patients with SCD. Therefore, the purpose of the study was to investigate gender differences in physical activity, sedentary behaviour, and aspects of physical fitness among Ghanaian adults with SCD. In addition, we examined the differences in outcome variables between patients with the haemoglobin SS and those with haemoglobin SC types of SCD. Associations between physical activity, sedentary time, body composition, flexibility and cardiovascular endurance were also determined. We hypothesized that male patients with SCD would exhibit higher physical activity and fitness than females.

## Methods

### Participants and setting

The participants of the study were patients who had been diagnosed as having SCD by a physician and were receiving medical treatment at the Ghana Institute of Clinical Genetics (Sickle Cell Clinic) of the Korle-Bu Teaching Hospital (KBTH) in Accra. Participants were included if they were aged 18-60 years, had been diagnosed as having SCD, and were willing to participate in the study. Individuals who had co-morbidities and physical impairments such as osteoarthritis that limited participation in assessment procedures were excluded. Also, patients who were in crisis and/or recovering from recent episodes of crisis were excluded. Ethical approval was granted by the Ethical and Protocol Review Committee of the School of Biomedical & Allied Health Sciences (SBAHS/10406071/AA/PHY/2015-2016). Permission was also sought from the management of the hospital and the head of the Institute. Each participant provided written informed consent before enrollment.

### Procedure

All the participants were tested in a designated cubicle at the clinic. All assessments were conducted over two visits by trained assessors (final year physiotherapy students) in the presence of a physician and nurse. On the first visit, anthropometric and demographic data were collected. Immediately after this, flexibility and cardiovascular endurance tests were administered. The participants were allowed 30 minutes rest between tests. On the second visit, the participant completed the physical activity questionnaire.

### Measures

### Anthropometric data

Height was assessed using a wall-mounted tape measure and weight was measured with a portable calibrated electronic scale. Body Mass Index (BMI) was computed by imputing the height and weight scores into the formula:

BMI=Weigh (kg)/height (m/^2^)

Demographic data such as age, gender, level of education and marital status were collected by self-report.

### The Sit-and-reach test

The sit-and-reach test (SRT) was used to measure flexibility. The test required participants to sit with their knees extended and the feet placed against a wooden box. They were instructed to place their right hand over the left and slowly reach forward as far as possible by sliding their hands along the box. The validity and reliability of the SRT have been previously reported [24-27]. The SRT was chosen because it is practical, less expensive and easy to administer. Three trials were conducted and the best score was used in the analysis.

### The three minute step test

The three minute step test (TMST) was used to evaluate cardiovascular endurance. The TMST was performed in accordance with recommended protocol [28]. Each participant was required to step on and off a 12inch wooden step 24 times for 3 minutes. A metronome set at 96 beats per minutes was used to synchronise the participants' stepping cycle [29]. After the test (within 1 minute), the participant sat on a chair and their heart rate was measured by auscultation using a stethoscope. All the participants completed the test (100%) without any adverse complaints.

### The Global Physical activity Questionnaire (GPAQ)

The Global physical activity questionnaire (GPAQ) was used to assess physical activity behavior [30]. The GPAQ is a 16-item scale that measures three major dimensions of physical activity; work, transport and recreational activities. The tool also evaluates sedentary behavior. Responses to the items of the questionnaire can be converted into component scores in metabolic equivalents (METs). Component scores are then summed to produce total physical activity which represents one's physical activity behavior per week. The tool has acceptable psychometric properties and is reported to be suitable for monitoring physical activity in adult populations [31, 32].

### Statistical analysis

The Shapiro-Wilk test was used to check for normality of the data. Descriptive data is presented as means, standard deviations and percentages. The independent sample t-test was used to determine mean differences of all continuous variables between males and females. Also, mean differences (of continuous variables) between patients with haemoglobin SS and those with haemoglobin SC were examined with the independent sample t-test. Since physical activity is related to physical fitness, we examined the relationship between physical activity, sedentary time, body composition and cardiovascular endurance using the Pearson's correlation coefficient. All statistical analyses were performed using the SPSS software version 23.0 at a significance level of p<0.05.

## Results

**Description of Participants' characteristics**. The descriptive characteristics of the participants are shown in Table 1. The majority of the participants were females 60.4% (32/53). The average age of the participants was (26.7±8.5years, range 18-52 years). The BMI of males and females were (20.4±2.0 kg/m^2^) and (21.0±3.9 kg/m^2^)) respectively. Most of the participants (52.8%) were identified as having the haemoglobin SS type of SCD Table 1.

**Table 1:** Sociodemographic characteristics of participants

Variables	Gender		Total (N=53)
	Males (n=21) (Mean±SD)	Females (n=32) (Mean±SD)	Total (N=53) (Mean±SD)
Age (years)	28.5±8.5	27.4±8.5	26.8±8.5
Height (m)	1.65±0.1	1.65±0.1	1.65±0.1
Weight (kg)	60.9±5.7	55.2±10.2	57.5±9.1
BMI (kg/m^2^)	20.4±2.0	21.0±3.9	20.8±3.3
**Level of education (n (%))**			
Tertiary education	9 (42.9)	16 (50)	25 (47.2)
Secondary education	10 (47.6)	12 (37.5)	22 (41.5)
Primary education	1 (4.8)	2 (6.3)	3 (5.7)
No formal education	1 (4.8)	1 (3.1)	1 (1.9)
**Marital status (n (%))**			
Married	4 (19)	8 (25)	12 (22.6)
Single	17 (81)	22 (68.8)	39 (73.6)
Widowed	0 (0)	2 (6.3)	2 (3.8)
**Sickle Cell Disease type (n (%))**			
Haemoglobin SS	9 (32.1)	19 (67.9)	28 (52.8)
Haemoglobin SC	13 (52.0)	12 (48.0)	25 (47.2)

### Comparisons of outcomes between males and females

As shown in Table 2, there were significant differences in total physical activity (t = 2.610, p = 0.012), moderate work-related physical activity (t = 3.587, p = 0.001) and moderate-recreational physical activity (t = 3.045, p = 0.004) between males and females. However, we found no statistically significant difference in sedentary time, flexibility, body composition and cardiovascular endurance between males and females Table 2.

**Table 2:** Comparison of physical activity, sedentary time, body composition, flexibility and cardiovascular endurance between males and females

Variables	Gender		Statistic	
	Male (n=21) (Mean±SD)	Females (n=32) (Mean±SD)	t(df=51)	p
**Physical activity**				
**Activity at work domain (average METs minutes per week)**				
Vigorous	33.3±107.6	0±0.00	1.762	0.084
Moderate	1834.45±1919.2	432.5±899.9	3.587	0.001
Travel to and from places domain (average METs minutes per week)	3329.5±3372.4	2629.4±2532.4	0.862	0.393
**Recreational activities domain (average METs minutes per week)**				
Vigorous	0±0.00	7.5±42.4	-0.807	0.423
Moderate	516.2±596.9	115.0±363.6	3.045	0.004
Total Physical activity (average METs minutes per week)	5713.5±4168.0	3184.4±2895.2	2.610	0.012
**Sedentary behaviour**				
Time spent in sedentary activities per day (minutes)	535.7±193.1	478.3±143.2	1.242	0.220
**Body composition**				
BMI (kg/m2)	20.4±2.0	21.0±3.9	-0.654	0.516
Flexibility				
Sit-and-reach test (cm)	5.6±2.2	4.6±2.6	1.325	0.191
**Cardiovascular endurance**				
Three-minute step test score (post-test heart rate in bpm)	121.2±20.4	124.4±15.8	-0.630	0.532

### Comparisons of outcomes between patients with haemoglobin SS and those with haemoglobin SC

We observed a significant difference in cardiovascular endurance (t = 2.640, p = 0.011) and body composition (t = -2.351, p = 0.023) between patients with haemoglobin SS and those with haemoglobin SC. There were no differences in physical activity, sedentary time and flexibility between the two patient groups Table 3.

**Table 3:** Comparison of physical activity, sedentary time, body composition, flexibility and cardiovascular endurance between patients with haemoglobin SS and those with haemoglobin SC

Variables	Sickle Cell Disease type		Statistic	
	Haemoglobin SS (n=28) (Mean±SD)	Haemoglobin SC (n=25) (Mean±SD)	t(df=51)	p
**Physical activity**				
**Activity at work domain (average METs minutes per week)**				
Vigorous	10.0±52.9	16.8±84.0	-0.357	0.723
Moderate	631.4±1075.3	1387.4±1881.2	-1.820	0.075
Travel to and from places domain (average METs minutes per week)	3297.9±3425.9	2468.8±2106.7	1.046	0.301
**Recreational activities domain (average METs minutes per week)**				
Vigorous	8.6±45.4	0±0.00	0.944	0.350
Moderate	242.9±477.2	308.8±542.2	-0.471	0.640
Total Physical activity (average METs minutes per week)	4190.7±3899.3	4181.8±3402.7	0.009	0.993
**Sedentary behaviour**				
Time spent in sedentary activities per day (minutes)	541.6±171.1	455.6±149.4	1.939	0.058
**Body composition**				
BMI (kg/m^2^)	19.8±2.9	21.8±3.5	-2.351	0.023
**Flexibility**				
Sit-and-reach test (cm)	4.9±2.9	5.1±2.0	-0.299	0.766
**Cardiovascular endurance**				
Three-minute step test, post-test heart rate (bpm)	128.9±19.4	116.7±13.1	2.640	0.011

### Associations between physical activities, sedentary time, body composition, flexibility and cardiovascular endurance

With regard to the relationship between the measured variables, we observed a moderately significant relationship between cardiovascular endurance and sedentary time (r = 0.437, p = 0.001). The relationship between cardiovascular endurance and any other variable was weak and non-significant Table 4.

**Table 4:** Correlations between physical activity, sedentary time, body composition, flexibility and cardiovascular endurance

Correlations	1	2	3	4	5
Total Physical activity (average METs in minutes per week)	-				
Sedentary behaviour, time spend in sedentary activities per day (minutes)	0.138	-			
BMI (kg/m^2^)	-0.079	-0.113	-		
Sit-and-reach test (cm)	0.040	0.039	-0.109	-	
Three-minute step test, post-test heart rate (bpm)	-0.253	0.437**	0.103	-0.033	-

**Abbreviations:** METs: Metabolic Equivalents, BMI: Body Mass Index, bpm: beats per minute, Note:

** : Correlation is significant at the 0.01 level (2-tailed). *: Correlation is significant at the 0.05 level (2-tailed)

## Discussion

In this study, SCD was found to be more prevalent in females compared to males. We also observed that the number of patients with the haemoglobin SS were more than those with haemoglobin SC type of SCD in our sample. In general, there was a significant difference in total physical activity, moderate work-related activity and moderate recreational activity between males and females. The males reported higher total physical activity levels than the females. In addition, there was a significant difference in cardiovascular endurance and body composition between patients with haemoglobin SS and those with haemoglobin. Finally, a moderately significant association was noted between cardiovascular endurance and sedentary time. Our finding that SCD is more common in females than males corroborates that of earlier research [23, 33]. Research in the field has often reported higher prevalence of SCD in females compared to males. The observations about the haemoglobin SS being the most common form of SCD in this population is also well supported in the literature [23].

The differences in physical activity between males and females may have several explanations. One possible explanation might be due to lack of motivation on the part of females to engage in physical activity. Usually females demonstrate aversion towards physical activity and this could be a major reason for the observed trend. Another possibility may be attributed to the stigma attached to the SCD in the Ghanaian culture. In Ghana, people with SCD are regarded as frail and incapable to handling stressful situations such as physical activity and exercise. This perception somewhat diminishes the confidence of patients with SCD and in particular women, making them to withdraw from physical activity and sports. Further, the majority of patients with SCD share the view that regular engagement in physical activity is detrimental to their health. In view of this, most patients especially females participate less in physical activity compared to the general population.

With regard to the types of SCD, differences in cardiovascular endurance and body composition was apparent between patients with haemoglobin SS and those with haemoglobin SC. It is unclear why these differences exist between the two groups. It can be argued that the types of SCD may have different impact on components of physical fitness. This could be related to the severity of their symptoms but since we did not assess disease severity, we are unable to explicitly justify this claim. Nonetheless, this finding would suggest that patients with SCD might exhibit different physical fitness profile depending on their sickling status. More research is needed to provide more illumination into the mechanism underlying this observation. The association between sedentary time and cardiovascular endurance may be explained by several mediating variables including musculoskeletal pain, frequency of hospitalization and poor quality of life. Patients who experience frequent painful episodes might spend more time in sedentary pursuits. The more time one spends in sedentary habits the longer it takes for their heart rate to return to normal levels following an exercise test. This is an indication that sedentary behaviour might be harmful to cardiovascular health. Therefore, it may be prudent for health professionals working with persons with SCD to educate them about the dangers of sedentariness.

There are several limitations of this study. First and foremost, the study involved a convenience sample of patients attending the Sickle cell clinic in KBTH. This might not be representative of the adult sickle cell population in Ghana. It is therefore useful for readers to be cautious not to generalize the results to patients in other geographical locations in the country. Secondly, we recruited 53 patients, a sample that is considered to be small in size. In addition the cross-sectional design does not allow for cause and effect relationships to be established. Another major limitation of the study lies in the instruments employed. The questionnaire used to measure physical activity allowed us to assess subjective physical activity behaviour. Questionnaire-based physical activity assessments have numerous challenges including the tendency for participants to either overestimate or underestimate their physical activity and sedentary time. Despite these limitations, to our knowledge this is the first study to document gender differences in physical activity among patients with SCD. Also, we have demonstrated the practicability of performing physical fitness assessments in this population. Future research should utilize objective measures of physical activity and a comprehensive battery of fitness tests to confirm these findings. Longitudinal data involving larger samples are needed to understand the interrelationship and stability of these factors over time.

## Conclusion

It can be concluded that physical activity levels of patients with SCD are different between males and females. Cardiovascular endurance and sedentary time are fairly related in this population. Longitudinal studies of objectively measured physical activity may provide better understanding of the mechanisms underlying sex differences in physical activity among adults with SCD.

### What is known about this topic

- Patients with SCD have reduced capacity to perform everyday activity tasks compared to their healthy peers;
- SCD is more common in females than males.

### What this study adds

- This is the first study to report on physical activity behavior in patients with SCD;
- Gender differences in physical activity are evident among patients with SCD.

## Competing interests

The authors declare no competing interests.
